# Association of diastolic and systolic blood pressure with depression: a cross-sectional study from NHANES 2005-2018

**DOI:** 10.3389/fpsyt.2024.1433990

**Published:** 2024-09-17

**Authors:** Huifeng Zhang, Ying Xu, Yaying Xu

**Affiliations:** ^1^ Department of Cardiovascular, The First Affiliated Hospital, and College of Clinical Medicine of Henan University of Science and Technology, Luoyang, China; ^2^ Department of Hematology, The First Affiliated Hospital, and College of Clinical Medicine of Henan University of Science and Technology, Luoyang, China; ^3^ Department of Endocrinology, The First Affiliated Hospital, and College of Clinical Medicine of Henan University of Science and Technology, Luoyang, China

**Keywords:** diastolic blood pressure, systolic blood pressure, depression, cross-sectional study, NHANES

## Abstract

**Background:**

Many studies worldwide have reported the association between mental health and blood pressure, but the results are mixed, and even contradictory. We aim to investigate the relationship between systolic and diastolic blood pressure and depression in the entire US population.

**Methods:**

This study analyzed cross-sectional data from the National Health and Nutrition Examination Survey (NHANES) from 2005 to 2018. All adults completed 3-4 blood pressure measurements after sitting quietly for 5 minutes. Depression was diagnosed based on the Patient Health Questionnaire (PHQ-9), with a score ≥10 defined as depression. Weighted logistic regression and restricted cubic splines (RCS) were used to assess the relationship between blood pressure and depression. Two-piecewise linear regression was used to determine the inflection point. Additionally, subgroup analyses and interaction tests were conducted to identify potential subgroups. Finally, two sensitivity analyses were conducted.

**Results:**

A total of 26,581 American adults were included, with a mean age of 47.2 years, of whom 13,354 (49.54%) were male; 2,261 individuals were defined as depressed, with a weighted prevalence of 7.41%. All participants’ mean systolic blood pressure (SBP) was 121.7 mmHg, and the mean diastolic blood pressure (DBP) was 70.9 mmHg. RCS showed a nonlinear association between SBP and depression, while DBP showed a positive linear association with depression. Two-piecewise linear regression showed that the inflection point of the association between SBP and depression was 129.7 mmHg. Weighted logistic regression showed that after fully adjusting for depression-related risk factors, there was a significant positive correlation between per 10 mmHg increase in DBP and depression (OR: 1.06, 95% CI: 1.00-1.12, P=0.04); however, only on the left side of the inflection point, SBP tended to decrease the odds of depression (P =0.09). Furthermore, interaction analysis showed that the association between DBP and depression was significantly stronger in cancer patients (P for interaction=0.02); on the left side of the inflection point (<129.7 mmHg), current smokers also significantly interacted with SBP (P for interaction=0.018). Finally, two sensitivity analyses also supported our findings.

**Conclusion:**

In the adult population of the United States, there is a positive linear association between DBP and depression, while the association between SBP and depression exhibits a significant threshold effect, maintaining SBP at 129.7 mmHg is associated with the lowest prevalence of depression.

## 1 Introduction

Depression is one of the most common psychological disorders and is the most important type among modern psychological disorders (1). Currently, one out of every twenty people may suffer from depression, and it is estimated that depression will become the leading burden of global diseases in five years (1, 2). In the United States, the prevalence of depression is higher among adults, with approximately one out of every seven people suffering from depression (3). Hypertension and emotional disorders often coexist and are considered as single or combined risk factors for cardiovascular disease (CVD) (4). The comorbidity of depression and CVD has long been a concern. As a maladaptive social disease, depression affects the occurrence and development of CVD, and CVD can also cause and exacerbate depression (5–7).

Blood pressure (BP) is a fundamental basis for the occurrence and development of CVD. Studies have shown that the association between SBP or DBP and depression has distinct characteristics, and the conclusions vary. While most studies support a negative association between SBP and depression (8–14); an observational study focusing on European-American individuals found no association between SBP and depression (15). The relationship between DBP and depression is complex, with evidence supporting no correlation (9, 15), positive correlation (16–21), and negative correlation (8, 10, 11, 13, 22). However, the possibility of non-linear association between BP and depression was rarely considered, and most studies did not comprehensively consider potential confounding factors or were limited by sample size, thereby affecting the external validity of their results. Therefore, this study comprehensively analyzed the association between DBP, SBP, and depression in a representative population of the United States using the NHANES database.

## 2 Methods

### 2.1 Study population

**Figure 1:**
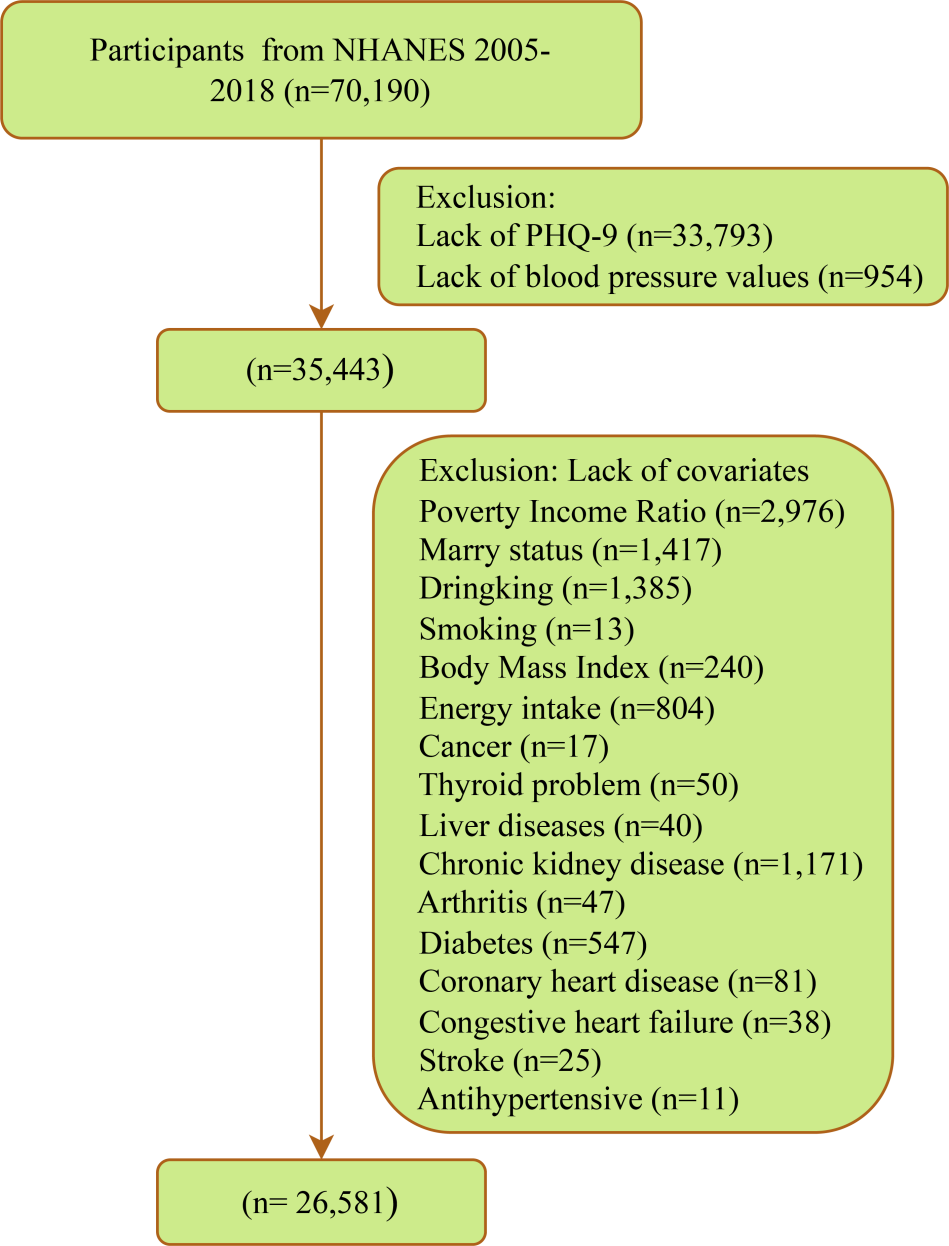
Flow chart for inclusion of participants.

This cross-sectional study investigated participants in 7 cycles of NHANES from 2005 to 2018. NHANES employs a complex multi-stage probability sampling design, involving data collection through face-to-face interviews, physical examinations, and laboratory tests, among other methods. The survey obtained ethical approval from the National Center for Health Statistics Ethics Review Board, and all participants provided written informed consent. Details are available at https://www.cdc.gov/nchs/nhanes/index.htm. A total of 70,190 participants were involved in 7 cycles, with exclusions made for 33,793 missing PHQ-9, 954 missing blood pressure measurements, and 8,862 missing covariates. Ultimately, 26,581 eligible participants were included (**Figure 1**).

### 2.2 Blood pressure (Exposure)

Blood pressure (BP) measurement, as part of physician examination within Mobile Examination Centers (MEC), applies to all participants aged 8 years and older. During examinations at Mobile Examination Centers (MEC) and in-home visits, all eligible individuals undergo three to four blood pressure measurements using mercury sphygmomanometers. Participants who are 50 years and older or less than one year of age who are unable to travel to the MEC are offered an abbreviated examination in their homes. The technique for obtaining blood pressure follows the recommendations of the American Heart Association for human blood pressure measurement (23). The calculation of systolic and diastolic blood pressure does not represent traditional averages but is computed according to the following protocol: if only one blood pressure reading was obtained, that reading is the average. If there is more than one blood pressure reading, the first reading is always excluded from the average. If only two blood pressure readings were obtained, the second blood pressure reading is the average. For more information on quality assurance and control, see Ostchega et al. (24).

### 2.3 Depression (Outcome)

Depression was determined based on the Patient Health Questionnaire-9 (PHQ-9), which assessed depressive symptoms present in the past two weeks. The sum of the individual scores for each question (0-3 points) constituted the depression score, with a total score ranging from 0 to 27 points, where higher scores indicated more severe depression. According to previous studies, a PHQ-9 total score ≥10 was defined as depression (25–27).

### 2.4 Covariates

In this study, the selection of covariates referred to the published works and the characteristics of the NHANES database. These confounding factors may have an impact on both BP and depression simultaneously (8, 10, 11).1) Demographic characteristics: age (<40 years, ≥60 years), sex (male/female), race (Mexican American, non-Hispanic black, non-Hispanic white, other Hispanic, other race—including multiracial), education level (less than college, college and above), marital status (divorced/separated/widowed, married/living with partner, never married); poverty income ratio (PIR) (<1.3, 1.3-3.5, >3.5). 2) Lifestyle habits and physical indicators: alcohol consumption (never, former, current), smoking (never, former, current), total dietary energy intake (classified into high and low groups based on median values), physical activity level (classified into high and low groups based on the median value of metabolic equivalent), and body mass index (BMI), which was defined as weight in kilograms divided by the square of height in meters (kg/m^2^). 3) Cardiovascular comorbidities: coronary heart disease (CHD), congestive heart failure (CHF), and stroke. 4) Other comorbidities: Cancer, thyroid disease, arthritis, liver disease, diabetes, chronic kidney disease (CKD) (diagnosed as CKD with A2, G3a, and above) (28), hyperlipidemia. 5) Medication history: Special consideration was given to antihypertensive and antidepressant medications.

### 2.5 Statistical analysis

The official documentation of NHANES recommends weighted data analysis (NHANES Tutorials - Weighting Module (cdc.gov)). In this study, the selected weight was the laboratory examination weight (1/7 * WTMEC2YR), as the key variables of this study were primarily obtained at the MEC. To account for the complex sampling methods involved in the NHANES database, Student’s t-test or Wilcoxon rank-sum test was used to compare continuous variables, with results presented as means (standard errors) or medians (interquartile ranges). Chi-square tests were used to analyze categorical variables, with results presented as counts (n) and percentages (%). Weighted logistic regression was used to estimate the association between exposure and outcome risk as odds ratios (ORs) and 95% confidence intervals (CIs). Multiple models were constructed: crude model, unadjusted; Model 1, adjusted for sex, age, race, and education level; Model 2, further adjusted for marital status, poverty-income ratio, alcohol consumption, smoking, energy intake, physical activity level, and BMI; Model 3, further adjusted for other comorbidities, including cancer, thyroid disease, arthritis, liver disease, diabetes, and chronic kidney disease; Model 4 further adjusted for cardiovascular comorbidities and medication history, including coronary artery disease, congestive heart failure, stroke, antihypertensive drugs, and antidepressants. Variance inflation factor (VIF) was used to assess collinearity, with all VIF values in this study being less than 10. If a nonlinear correlation is presented, according to previous studies, the classified DBP is defined with cut-off values of 60 mmHg, 80 mmHg, and 90 mmHg, and the classified SBP is defined with cut-off values of 90 mmHg, 120 mmHg, 130 mmHg, and 140 mmHg (8, 29, 30). In models with categorical independent variables, the median of each group was included in the logistic regression model to test the trend effect of the association between BP and depression. Subgroup analyses were conducted, and likelihood ratio tests were used to explore interactions between covariates and blood pressure. Restricted cubic spline (RCS) with three knots (10th, 50th, and 90th percentiles) was used to evaluate the exposure-dose relationship between continuous BP and depression based on the principle of minimizing the Akaike information criterion (AIC). For nonlinear associations, two-piecewise linear regression was used to determine inflection points. In addition, two sensitivity analyses were performed: 1) without considering sampling weights; 2) multiple imputation for covariates with missing proportions below 10%.

All statistical procedures in this study were conducted using R software version 4.3.3 (R Foundation for Statistical Computing). Weighted regression analysis was performed using the “survey” package; RCS regression was constructed using the “rms” package; segmented regression was fitted using the “segmented” package to determine inflection points; VIF was calculated using the “car” package; multiple imputation was conducted using the “mice” package. In this study, two-sided p-values <0.05 were considered statistically significant.

## 3 Results

### 3.1 Population characteristics

After strict screening, a total of 26,581 participants with complete data were included (**Figure 1**). As shown in **Table 1**, the mean age of all participants was 47.2 years, with 13,354 males (49.54%); 2,261 individuals were defined as depressed, with a weighted prevalence of 7.41%; the mean SBP for all participants was 121.7 mmHg, and the mean DBP was 70.9 mmHg. Compared to the non-depressed group, depressed patients were more likely to be female, under 60 years old, non-Hispanic black, have lower education levels, be never married or divorced/separated/widowed, be impoverished, have a history of alcohol consumption, currently smoke, and be overweight or obese; they also had lower energy intake and lower levels of physical activity. Moreover, individuals currently suffering from depression were more likely to have several cardiovascular comorbidities (CHD, CHF, and stroke) and other comorbidities (arthritis, thyroid problems, liver issues, DM, CKD, and hyperlipidemia). Additionally, the use of antidepressants or antihypertensive drugs in the past month was more common among the depressed population.

**Table 1:** Weighted characteristics of the eligible 26,581 participants.

Characteristics	Total (N=26,581)	Without depression (N=24,320)	Depression (N=2,261)	P value
Diastolic blood pressure, Mean (SE)	70.89(0.19)	70.85(0.20)	71.43(0.31)	0.07
Systolic blood pressure, Mean (SE)	121.73(0.18)	121.75(0.19)	121.45(0.48)	0.54
Sex, n (%)				< 0.0001
Female	13227(50.46)	11807(49.45)	1420(63.10)	
Male	13354(49.54)	12513(50.55)	841(36.90)	
Age, Mean (SE)	47.20(0.26)	47.27(0.27)	46.35(0.44)	0.06
Age, n (%)				< 0.001
<60 years	17911(74.84)	16260(74.46)	1651(79.57)	
≥60 years	8670(25.16)	8060(25.54)	610(20.43)	
Race, n (%)				< 0.0001
Mexican American	4064(7.93)	3738(7.97)	326(7.36)	
Non-Hispanic Black	5436(10.11)	4957(9.90)	479(12.72)	
Non-Hispanic White	12073(70.28)	11055(70.62)	1018(65.92)	
Other Hispanic	2406(5.00)	2129(4.84)	277(7.00)	
Other Race - Including Multi-Racial	2602(6.69)	2441(6.66)	161(6.99)	
Education attainment, n (%)				< 0.0001
Less than college	12126(37.44)	10809(36.35)	1317(51.10)	
College or higher	14455(62.56)	13511(63.65)	944(48.90)	
Marital status, n (%)				< 0.0001
Never married	4734(17.49)	4268(17.22)	466(20.86)	
Divorced/separated/widowed	5802(18.10)	5033(17.11)	769(30.51)	
Married/living with a partner	16045(64.42)	15019(65.68)	1026(48.63)	
PIR, n (%)				< 0.0001
<1.3	7993(19.86)	6820(18.22)	1173(40.47)	
1.3–3.5	10051(35.46)	9297(35.43)	754(35.83)	
≥3.5	8537(44.68)	8203(46.36)	334(23.70)	
Drinking status, n (%)				< 0.0001
Never	3512(10.12)	3243(10.22)	269(8.77)	
Former	4300(13.12)	3812(12.63)	488(19.34)	
Now	18769(76.76)	17265(77.15)	1504(71.89)	
Smoking status, n (%)				< 0.0001
Never	14492(54.81)	13597(56.15)	895(38.04)	
Former	6565(25.15)	6052(25.32)	513(22.94)	
Now	5524(20.05)	4671(18.53)	853(39.02)	
Energy intake, n (%)				< 0.0001
Low	13284(46.25)	12037(45.75)	1247(52.53)	
High	13297(53.75)	12283(54.25)	1014(47.47)	
BMI, n (%)				0.01
<25Kg/m^2^	7601(29.84)	7058(30.14)	543(26.00)	
≥25Kg/m^2^	18980(70.16)	17262(69.86)	1718(74.00)	
MET level, n (%)				< 0.0001
LOW	10011(39.29)	9252(39.67)	759(34.53)	
High	10036(40.61)	9343(41.21)	693(33.09)	
Unknown	6534(20.11)	5725(19.12)	809(32.38)	
Arthritis, n (%)	7216(25.62)	6206(24.27)	1010(42.41)	< 0.0001
Cancer, n (%)	2513(9.91)	2261(9.79)	252(11.46)	0.09
Thyroid problem, n (%)	2715(10.84)	2359(10.38)	356(16.58)	< 0.0001
Liver problem, n (%)	1021(3.43)	836(3.10)	185(7.48)	< 0.0001
DM, n (%)	4858(13.62)	4272(13.09)	586(20.20)	< 0.0001
CHD, n (%)	1046(3.27)	903(3.10)	143(5.38)	< 0.0001
CHF, n (%)	794(2.19)	641(1.93)	153(5.38)	< 0.0001
CKD, n (%)	4734(14.06)	4244(13.87)	490(16.49)	0.001
Stroke, n (%)	978(2.74)	800(2.44)	178(6.46)	< 0.0001
Hyperlipidemia, n (%)	18933(70.28)	17204(69.90)	1729(75.14)	< 0.001
Antihypertensive drug, n (%)	8292(26.79)	7441(26.20)	851(34.25)	< 0.0001
Antidepressants, n (%)	2868(12.87)	2140(10.97)	728(36.65)	< 0.0001

MET, Metabolic Equivalent; PIR, poverty income ratio; BMI, body mass index; DM, diabetes; CHD, coronary heart disease; CHF, congestive heart failure; CKD, chronic kidney disease; OR, odds ratio; CI, confidence interval; SE, standard error.

### 3.2 Nonlinear analysis of continuous BP with prevalent depression and determination of inflection point

**Figure 2:**
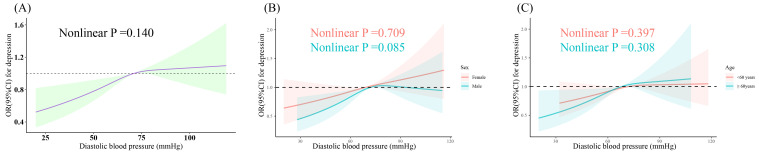
Dose-response relationship between DBP and prevalent depression. Three knots (10th, 50th, 90th percentiles) were selected for fitting the restricted cubic spline model, and the median value of SBP (121 mmHg) and DBP (71 mmHg) served as the reference point. All models were adjusted for age, sex, ethnicity, education, marital status, poverty-income ratio, total energy intake, BMI, physical activity level, smoking, drinking, arthritis, thyroid problems, cancer, liver problems diabetes, DM, CHD, CHF, CKD, stroke, hyperlipidemia, antihypertensive drug, and antidepressant. SBP, systolic blood pressure; DBP, diastolic blood pressure; BMI, body mass index; DM, diabetes; CHD, coronary heart disease; CHF, congestive heart failure; CKD, chronic kidney disease; OR, odds ratio; CI, confidence interval. **(A)** Dose-response relationship between DBP and depression in the overall population. **(B)** Dose-response relationship between DBP and depression in different gender subgroups. **(C)** Dose-response relationship between DBP and depression in different age subgroups.

**Figure 3:**
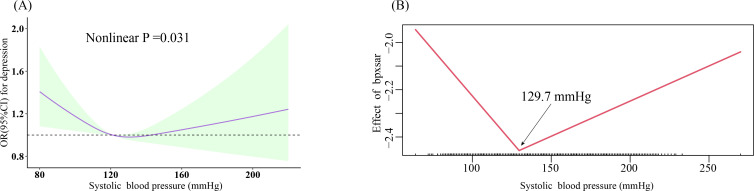
Dose-response relationship and the two-piecewise linear regression of SBP with prevalent depression. **(A)** Restricted cubic spline; three knots (10th, 50th, 90th percentiles) were selected for fitting the restricted cubic spline model, and the median value of SBP (121 mmHg) and DBP (71 mmHg) served as the reference point; **(B)** two-piecewise linear regression. All models were adjusted for age, sex, ethnicity, education, marital status, poverty-income ratio, total energy intake, BMI, physical activity level, smoking, drinking, arthritis, thyroid problems, cancer, liver problems diabetes, DM, CHD, CHF, CKD, stroke, hyperlipidemia, antihypertensive drug, and antidepressant. SBP, systolic blood pressure; DBP, diastolic blood pressure; BMI, body mass index; DM, diabetes; CHD, coronary heart disease; CHF, congestive heart failure; CKD, chronic kidney disease; OR, odds ratio; CI, confidence interval.

The fully adjusted RCS displayed a positive linear association between DBP and depression (nonlinear P =0.140) (**Figure 2A**), which remained consistent after stratification by sex and age (all nonlinear P >0.05, respectively) (**Figures 2B, C**). SBP exhibited a U-shaped association with depression (nonlinear P =0.031) (**Figure 3A**); two-piecewise linear regression indicated a turning point of 129.7 mmHg for the association between SBP and depression (**Figure 3B**).

### 3.3 Association of per-10 increase in BP with prevalent depression

**Table 2** displays the weighted logistic regression results for the association between BP and prevalent depression. When not considering confounding factors (crude model), there was no significant association between DBP and depression (OR: 1.05, 95% CI: 1.00-1.10, P =0.07); after adjusting for sex, age, race, and education level (model 1), we estimated that the odds ratios (ORs) of depression associated with a per 10-unit increase in DBP were 1.07 (95% CI: 1.01-1.13, P = 0.01); after full adjustment (model 4), the association between depression and DBP was somewhat attenuated but still significant (OR: 1.06, 95% CI: 1.00-1.12, P =0.04). After categorizing DBP into four groups and fully adjusting (model 4), compared with the <60 mmHg group, the odds of depression increased by 27% in the 60-79 mmHg group (OR: 1.27, 95% CI: 1.04-1.55, P =0.02) and by 34% in the 80-89 mmHg group (OR: 1.34, 95% CI: 1.03-1.72, P =0.03); although there was no significant change in depression odds in the ≥90 mmHg group (OR: 1.27, 95% CI: 0.95-1.69, P =0.10), there was a statistically significant trend among these four categories (p trend=0.03). **Table 2** also presents the weighted logistic regression results for the association between SBP and depression. Our results indicate that overall, SBP was not significantly associated with depression (OR: 0.97, 95% CI: 0.94-1.01, P =0.11); when SBP was ≥129.7 mmHg, there was also no association with depression (OR: 1.02, 95% CI: 0.94-1.11, P =0.57); SBP at <129.7 mmHg tended to reduce the odds of depression (OR: 0.93, 95% CI: 0.86-1.01, P =0.09).

**Table 2:** Weighted multivariate Logistic regression analysis for the association of prevalent depression with SBP and DBP.

	Crude model		Model 1			Model 2			Model 3		Model 4	
	95%CI	P	95%CI		P	95%CI		P	95%CI	P	95%CI	P
Continuous DBP (per 10 mmHg+)	1.05(1.00,1.10)	0.07	1.07(1.01,1.13)		0.01	1.08(1.02,1.14)		0.01	1.07(1.01,1.13)	0.02	1.06(1.00,1.12)	0.04
DBP <60	ref		ref			ref			ref		ref	
60 ≤DBP≤ 79	1.09(0.91,1.30)	0.36	1.11(0.93,1.33)		0.24	1.25(1.04,1.51)		0.02	1.25(1.03,1.53)	0.02	1.27(1.04,1.55)	0.02
80 ≤DBP≤ 89	1.19(0.96,1.48)	0.10	1.27(1.02,1.58)		0.03	1.42(1.12,1.80)		0.004	1.36(1.06,1.74)	0.02	1.34(1.03,1.72)	0.03
90 ≤DBP	1.24(0.95,1.62)	0.12	1.34(1.02,1.76)		0.04	1.34(1.02,1.76)		0.04	1.29(0.96,1.72)	0.09	1.27(0.95,1.69)	0.10
p for trend		0.04			0.01			0.002		0.01		0.03
Continuous SBP (per 10 mmHg+)	0.99(0.96,1.02)	0.55	1.02(0.98,1.05)		0.33	0.99(0.96,1.03)		0.70	0.97(0.94,1.01)	0.12	0.97(0.94,1.01)	0.11
SBP <129.7	0.92(0.85,0.99)	0.04	0.98(0.91,1.07)		0.69	0.97(0.90,1.05)		0.49	0.95(0.88,1.03)	0.21	0.93(0.86,1.01)	0.09
SBP ≥129.7	1.05(0.97,1.14)	0.21	1.05(0.97,1.13)		0.20	1.00(0.93,1.08)		0.91	1.01(0.94,1.09)	0.81	1.02(0.94,1.11)	0.57

Crude model: Not adjusted.

Model 1: Adjusted for age, sex, education attainment, and ethnicity.

Model 2: Further adjusted for marital status, poverty-income ratio, smoking, and drinking status, BMI, physical activity level, and total energy intake based on Model 1.

Model 3: Further adjusted for arthritis, thyroid problems, cancer, diabetes, and liver diseases based on Model 2.

Model 4: Further adjusted for CHD, CHF, CKD, stroke, hyperlipidemia, antihypertensive drug, and antidepressant based on Model 3.

SBP, systolic blood pressure; DBP, diastolic blood pressure; BMI, body mass index; CHD, coronary heart disease; CHF, congestive heart failure; CKD, chronic kidney disease; OR, odds ratio; CI, confidence interval.

### 3.4 Subgroup analysis and effect modification tests

**Figure 4:**
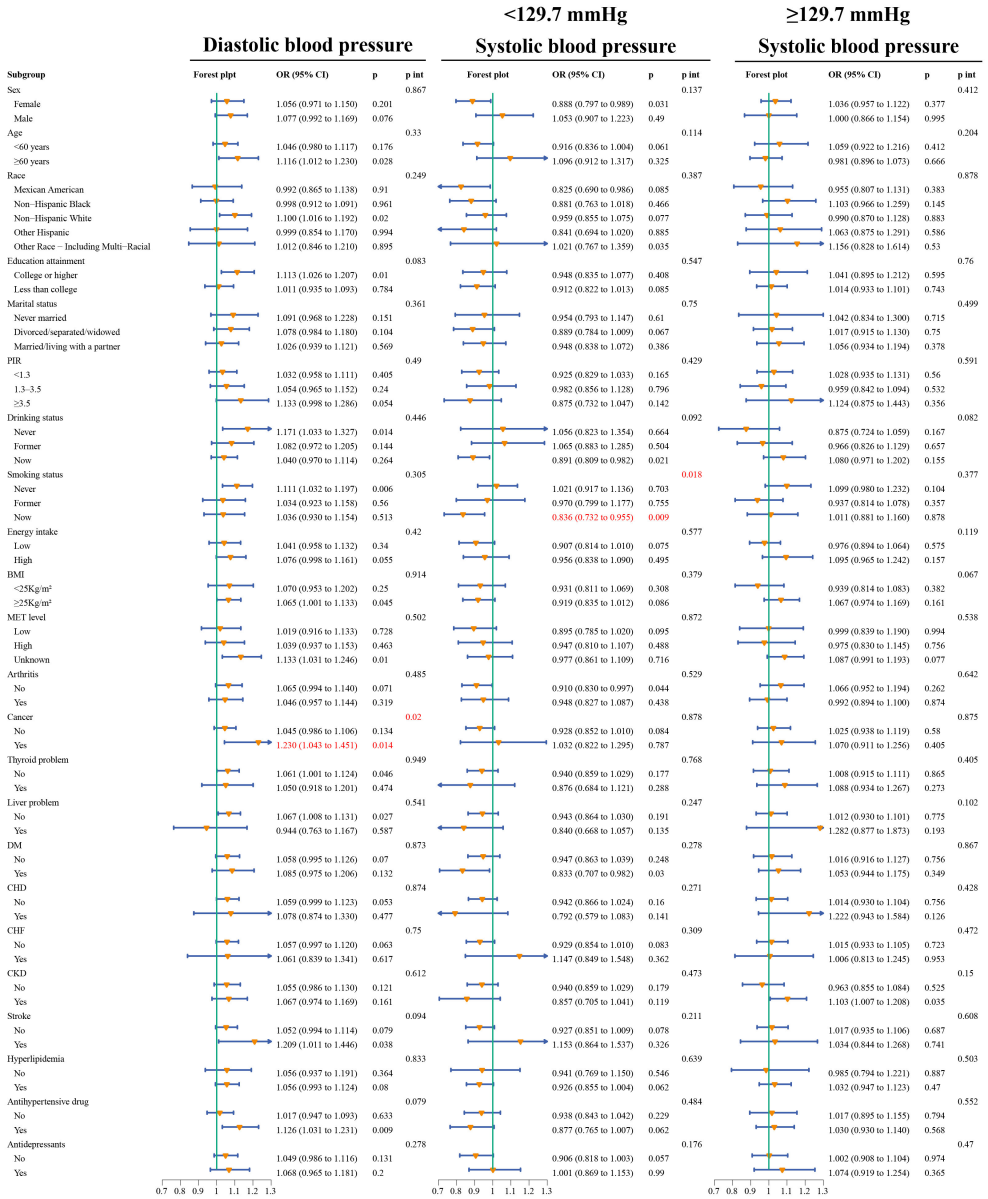
Forest plot of subgroup analysis and interaction tests for the association of SBP and DBP with the odds of prevalent depression. All models adjusted for 22 risk factors other than stratification variables, and the significance of the interaction was determined by the likelihood ratio test. The stratified logistic regression model also considers sample weights. PIR, poverty income ratio; MET, metabolic equivalent; BMI, body mass index; DM, diabetes; CHD, coronary heart disease; CHF, congestive heart failure; CKD, chronic kidney disease; OR, odds ratio; CI, confidence interval; p int, p for interaction.

**Figure 4** illustrates the association between depression and BP across various subgroups. Interaction analysis reveals that among cancer patients, the association between SBP and depression was significantly stronger (P for interaction=0.02); furthermore, on the left side of the inflection point (<129.7 mmHg), current smokers exhibited a more pronounced decrease in the likelihood of depression as blood pressure increases (P for interaction=0.018).

### 3.5 Sensitivity analysis

As shown in **Table 3**, our findings were still supported after disregarding weights or after multiple interpolation for missing covariates.

**Table 3:** Sensitivity analysis for the association of prevalent depression with SBP and DBP.

		Crude model		Model 1			Model 2			Model 3		Model 4	
		95%CI	P	95%CI		P	95%CI		P	95%CI	P	95%CI	P
Without weighted (sensitivity analysis 1)	Continuous DBP (per 10 mmHg+)	1.04(1.00,1.08)	0.05	1.06(1.02,1.10)		0.003	1.06(1.02,1.10)		0.002	1.05(1.01,1.09)	0.01	1.05(1.01,1.09)	0.02
	DBP <60	ref		ref			ref			ref		ref	
	60 ≤DBP≤ 79	1.04(0.93,1.18)	0.49	1.05(0.93,1.19)		0.40	1.14(1.01,1.30)		0.04	1.14(1.00,1.30)	0.04	1.14(1.00,1.30)	0.05
	80 ≤DBP≤ 89	1.10(0.94,1.28)	0.24	1.15(0.99,1.35)		0.07	1.23(1.05,1.45)		0.01	1.19(1.01,1.41)	0.04	1.17(0.99,1.39)	0.07
	90 ≤DBP	1.17(0.95,1.44)	0.13	1.28(1.03,1.58)		0.02	1.25(1.00,1.55)		0.05	1.23(0.98,1.53)	0.07	1.22(0.97,1.53)	0.08
	p for trend		0.09			0.01			0.01		0.03		0.05
	Continuous SBP (per 10 mmHg+)	0.98(0.96,1.01)	0.20	1.02(0.99,1.04)		0.20	1.00(0.97,1.02)		0.76	0.98(0.95,1.00)	0.09	0.98(0.95,1.00)	0.11
	SBP <129.7 (per 10 mmHg+)	0.91(0.86,0.96)	<0.001	0.97(0.92,1.03)		0.28	0.95(0.90,1.01)		0.12	0.93(0.88,0.99)	0.01	0.92(0.87,0.98)	0.01
	SBP ≥129.7 (per 10 mmHg+)	1.05(0.99,1.10)	0.07	1.05(1.00,1.11)		0.07	1.02(0.97,1.08)		0.47	1.03(0.97,1.08)	0.33	1.03(0.97,1.09)	0.29
Weighted after multiple interpolation (sensitivity analysis 2)	Continuous DBP (per 10 mmHg+)	1.05(1.00,1.09)	0.03	1.08(1.04,1.13)		<0.001	1.09(1.04,1.14)		<0.001	1.07(1.02,1.12)	0.004	1.06(1.01,1.11)	0.01
	DBP <60	ref		ref			ref			ref		ref	
	60 ≤DBP≤ 79	1.05(0.91,1.22)	0.49	1.12(0.96,1.30)		0.14	1.22(1.05,1.43)		0.01	1.21(1.03,1.42)	0.02	1.21(1.03,1.42)	0.02
	80 ≤DBP≤ 89	1.16(0.96,1.40)	0.12	1.29(1.07,1.56)		0.01	1.39(1.14,1.69)		0.001	1.32(1.08,1.62)	0.01	1.29(1.05,1.58)	0.02
	90 ≤DBP	1.29(1.03,1.61)	0.03	1.46(1.17,1.82)		0.001	1.40(1.12,1.76)		0.004	1.30(1.02,1.66)	0.03	1.26(0.99,1.61)	0.06
	p for trend		0.02			<0.001			<0.001		0.004		0.01
	Continuous SBP (per 10 mmHg+)	1.00(0.97,1.03)	0.81	1.02(0.99,1.06)		0.16	1.00(0.97,1.03)		0.82	0.97(0.94,1.00)	0.09	0.97(0.94,1.00)	0.08
	SBP <129.7 (per 10 mmHg+)	0.92(0.87,0.98)	0.01	0.99(0.92,1.06)		0.72	0.97(0.91,1.04)		0.44	0.95(0.89,1.02)	0.13	0.93(0.87,1.00)	0.06
	SBP ≥129.7 (per 10 mmHg+)	1.05(0.99,1.12)	0.12	1.04(0.98,1.11)		0.18	1.00(0.93,1.06)		0.93	1.00(0.94,1.07)	0.97	1.00(0.94,1.07)	0.96

Crude model: Not adjusted;

Model 1: Adjusted for age, sex, education attainment, and ethnicity;

Model 2: Further adjusted for marital status, poverty-income ratio, smoking, and drinking status, BMI, physical activity level, and total energy intake based on Model 1;

Model 3: Further adjusted for arthritis, thyroid problems, cancer, diabetes, and liver diseases based on Model 2;

Model 4: Further adjusted for CHD, CHF, CKD, stroke, hyperlipidemia, antihypertensive drug, and antidepressant based on Model 3;

SBP, systolic blood pressure; DBP, diastolic blood pressure; BMI, body mass index; CHD, coronary heart disease; CHF, congestive heart failure; CKD, chronic kidney disease; OR, odds ratio; CI, confidence interval.

## 4 Discussion

In this nationally representative study, our results demonstrate that DBP showed an almost linear increase in the odds of depression, while SBP exhibited a U-shaped association with prevalent depression, with an inflection point at 129.7 mmHg. Below this inflection point, increasing SBP tended to lower the odds of depression, whereas above it, the increase in SBP was not associated with depression. Even when not considering weighting or conducting multiple imputations for covariates with missing proportions less than 10%, the results remained robust. Additionally, through interaction analysis, we identified significant modification effects of cancer on the association between DBP and depression, indicating that cancer patients had a greater likelihood of depression with increasing DBP. Furthermore, a stronger association between SBP and depression was observed among current smokers compared to non-smokers or former smokers when SBP was <129.7 mmHg.

Based on our knowledge, this is the first cross-sectional study conducted on a nationally representative population, investigating the association between mean multiple BP measurements and the odds of depression. Despite extensive discussions on the association between BP and depression worldwide, the conclusions remain highly controversial. Most studies support a negative association between SBP and depression (8–14); in our study, when SBP was below 129.7 mmHg, the odds of depression tended to decrease with increasing SBP. However, an observational study focusing on European Americans found no association between SBP and depression (15); our results indicate no significant association between SBP and prevalent depression when SBP was greater than 129.7 mmHg. Previous research has seldom explored the nonlinear association between SBP and depression, a hypothesis confirmed in our research. On the other hand, the relationship between DBP and depression is complex, with evidence supporting no association (9, 15), positive association (16–21), and negative association (8, 10, 11, 13, 22). Consistent with most studies, our results support a positive association between DBP and prevalent depression.

Previous studies have actually presented a paradox: if SBP is inversely correlated with depression, this implies that higher SBP appears to be associated with lower depression risk. In 2023, Schaare et al. (31) provided insightful insights into this seemingly contradictory phenomenon. Through analyzing extensive psychological, medical, and neuroimaging data from the UK Biobank, their research team found that higher SBP was associated with fewer depressive symptoms and greater happiness. However, being diagnosed with hypertension was associated with more depressive symptoms and lower happiness. Our study is highly consistent with this finding, showing an inverse association trend between SBP on both sides of 129.7 mmHg and the odds of depression. Mechanistically, although the causal pathways between BP and mental health are not fully understood and may involve multiple factors, arterial blood pressure may serve as an important bridge to the central regulation circuits of the brain. Activation of baroreceptors influences emotion and pain processing, thereby modulating the behavioral and central effects of BP changes (32–34). Moderate increases in SBP may lead to gradual desensitization of baroreceptors and alterations in sensory processing, which could gradually adjust pain sensitivity thresholds, alter sensory and emotional processing, reduce cortical excitability, and suppress central nervous system activity (35–37). Therefore, relatively low-sensitive baroreceptor signals may form the basis for the lower depression risk in populations with SBP at 129.7 mmHg. However, the hazards of other chronic conditions associated with elevated SBP remain a serious medical issue. Research suggests that the burden of hypertension and other vascular risk factors may lead to microvascular brain damage, thereby causing depressive symptoms (38). Additionally, our study also suggests that the odds of depression risk decrease more significantly with moderate increases in SBP among current smokers. Smoking induces various physiological responses, including vasoconstriction, increased heart rate, and changes in chemical composition in the blood, which may affect SBP levels and indirectly influence the occurrence of depressive symptoms (39). Smoking itself is closely associated with mental health issues, as smokers may be more prone to experiencing negative emotions such as anxiety and depression. These psychosocial factors may interact with the negative correlation between SBP and depression, thereby exacerbating the relationship between the two (40). Furthermore, smokers may experience temporary feelings of tranquility and relaxation through inhaling components such as nicotine, which may temporarily alleviate their depressive symptoms. This pharmacological effect may lead to some degree of relief in depressive symptoms among smokers while also potentially affecting SBP levels (41–43).

Six previous studies supported a positive association between DBP and the likelihood of depression (16–21). However, these studies focused on specific populations, such as medical students, children, police officers, or rural populations. Our study has universality. Common risk factors or shared genetic influences may be important pathways linking elevated DBP to depression (44, 45). Barinas-Mitchell et al. (19) showed that individuals who had depression during childhood and their siblings had higher DBP compared to the control group. In turn, Olive et al. (20) tracked the relationship between dynamic changes in DBP from childhood to adolescence and depressive symptoms, finding that depressive symptoms led to an increase in DBP, which had some impact on the subsequent risk of CVD. Additionally, our results also showed that cancer enhanced the association between DBP and depression. The modifying effect of cancer may involve multiple factors such as biological, psychosocial, and therapeutic aspects (46–49). Further research can explore how these factors interact to better understand the impact of cancer on mental health and cardiovascular health.

## 5 Advantages and limitations

The large sample size and representation of 167 million U.S. adults weighted in this study were significant advantages. Additionally, we constructed multiple models, comprehensively considering confounding factors that simultaneously influence depression and blood pressure. Furthermore, our conclusions are relatively robust; even without considering weights, the conclusions remain largely consistent. Moreover, we addressed potential overall bias due to covariate missingness, further validating the robustness of our conclusions through sensitivity analysis using multiple imputation. Besides, as emphasized by Lim et al. (8), further research is needed to establish the optimal blood pressure range to prevent incident depression and reduce hypertension complications. Our study provides reference values for SBP, determining through nonlinear analysis that the odds of depression are lowest at an SBP of 129.7 mmHg, thus addressing the paradox of the relationship between BP and depression.

However, several limitations need to be noted. Firstly, due to its cross-sectional design, causality between depression and BP cannot be inferred, necessitating further validation in larger prospective cohort studies. Secondly, the diagnosis of depression in this study was based on self-reporting, which may be inaccurate and cannot be generalized to the true incidence of clinical depression. Thirdly, despite the high scientific rigor of the measurement and statistical methods provided by NHANES, BP values are inevitably influenced by many genetic and environmental factors. Fourthly, despite comprehensive consideration of covariates that simultaneously affect BP and depression, potential confounding factors are inevitably overlooked. Fifthly, on the left side of the inflection point, SBP shows a marginally significantly association with depression. In the sensitivity analysis, the P values are 0.01 and 0.06 respectively. Therefore, we cautiously interpret that when SBP is less than 129.7 mmHg, SBP tends to have a negative association with depression. Finally, the limitation of this study lies in the sample being limited to the U.S. population, thus requiring caution in interpreting the external validity of the study results.

## 6 Conclusion

In conclusion, our study provides a new clinical perspective and evidence on the relationship between BP and depression. DBP was positively linearly associated with prevalent depression, while the association between SBP and the odds of depression exhibits a threshold effect, with a turning point at 129.7 mmHg.

## Data Availability

Publicly available datasets were analyzed in this study. This data can be found here: https://www.cdc.gov/nchs/nhanes/index.htm.
